# Is health related quality of life influenced by diabetic neuropathic pain among type II diabetes mellitus patients in Ethiopia?

**DOI:** 10.1371/journal.pone.0211449

**Published:** 2019-02-04

**Authors:** Hiwot Degu, Abigiya Wondimagegnehu, Yared Mamushet Yifru, Ayele Belachew

**Affiliations:** 1 Department of Preventive Medicine, School of Public Health, Addis Ababa University, Addis Ababa, Ethiopia; 2 Department of Neurology, School of Medicine, Addis Ababa University, Addis Ababa, Ethiopia; Weill Cornell Medicine-Qatar, QATAR

## Abstract

**Background:**

Polyneuropathy is one of the commonest complications of long-standing diabetes. Progressive sensory loss can predispose patients to foot ulcer and the neuropathy oftentimes causes pain. The pain can significantly affect the quality of life of patients.

**Objectives:**

To describes the health-related quality of life of patients with type II diabetes mellitus suffering from painful diabetic peripheral neuropathy at two referral hospitals in Addis Ababa, Ethiopia, 2017.

**Methods:**

An institution based cross sectional study with internal comparison was conducted among a sample of 220 type II diabetes mellitus patients in a 1:1 matched ratio of those with and without diabetes associated peripheral neuropathic pain. All were having regular follow up at two hospitals in Addis Ababa, Ethiopia. The Short Form (SF-36) health-related quality of life instrument was used to collect data on quality of life while basic socio-demographic and other disease specific features were collected using a structured questionnaire. Descriptive statistics was used to examine the mean scores of health related quality of life. Cronbach’s alpha coefficient and Pearson’s correlation coefficient were applied to estimate the internal consistency, and the level of agreement between the different domains of SF-36, respectively. To measure association between health related quality of life domains and explanatory variables, independent T-test and ANOVA were performed followed by multiple linear regression analyses.

**Results:**

The health related quality of life of type II diabetes mellitus patients with peripheral neuropathic pain was poorer than those without pain in all the eight domains and the two summary scores by SF-36 (p < 0.001). Higher mean score difference was observed in Mental Component Summary Score (MCS) (14.6) compared to Physical Component Score (PCS) (9.3). Among the eight domains, the largest mean difference was found with the physical one (39.1) followed by mental health (38.2) and physical functioning (30). Pain intensity had a statistically significant negative correlation with all domains as well as the two summary scores. Younger age, a higher level of education, being single, a higher monthly income, normal body mass index, HbA1c less than seven mmo/L, absence of other diabetic complications and taking only oral hypoglycemic agents were found to predict better health related quality of life.

**Conclusion:**

The presence of diabetic peripheral neuropathic pain was found to negatively influence the health-related quality of life of type II diabetic patients; the greatest impact being on the ‘role physical’ and ‘mental health’ domains. Older age, presence of diabetes related complications, longer duration of illness negatively influenced the health-related quality of life.

## Introduction

Diabetes mellitus is one of the most common chronic diseases that affect people of all ages and races worldwide. The magnitude of Diabetes Mellitus (DM) continues to increase mainly due to changes in lifestyles resulting in physical inactivity and increased obesity. Globally 6% of adults are estimated to have either DM I or II, and of these 80% live in low and middle-income countries. If this trend continues, an estimated 592 million people, or one in ten adults will have diabetes by 2035 [1]. In Africa, diabetes is estimated to affect about 19.8 million adults; and in Ethiopia alone, 1.9 million patients are believed to exist [2]. In 2015, diabetes was responsible for about 6% of adult deaths in the African Region [2]. According to International Diabetes Federation (IDF) 2015 report, among the Ethiopian total adult population, 1.3 million had DM. According to the WHO estimates, 90% diabetic patients have type II diabetes [2].

One of the commonest complications of type II DM is Diabetic Peripheral Neuropathy (DPN) which leads to a substantial reduction in the quality of life [3–6]. DPN is defined as the presence of peripheral nerve dysfunction in diabetics after exclusion of other causes [5]. DPN could result in complications like foot ulcer, infections and non-traumatic amputation [7]. Peripheral neuropathy may be symptomatic or asymptomatic. When symptoms are present, they may be negative or positive; negative symptoms include loss of sensation and loss of strength, while positive symptoms include pricking sensation or pain. Among patients with DPN, 11% could have pain called Diabetic Peripheral Neuropathic Pain (DPNP). Clinically, DPNP could present with pain of burning, shooting or aching type. It may also be accompanied by allodynia, hyperalgesia and numbness which often get worse at night that could result in disturbed sleep, anxiety and depression. The sleep deprivation leads to lack of energy, strongly influencing patient’s ability to function from decreased mobility and dependence on others in everyday functioning [7,8]. DPNP can have a profound effect on one’s quality of life in terms of social and psychological wellbeing, as well as physical ill-health, and is one of the most psychologically debilitating symptoms of the disease [8, 9].

The prevalence of DPN in Van Acker et al study was 43%, and higher in type II diabetes (50.8%) compared with type I (25.6%). The study also documented 14% prevalence of PDPN which again was higher in type II (17.9%) compared with type 1 (5.8%) patients. The prevalence increased with the duration of diabetes and age of the patient. Nephropathy, obesity, low HDL cholesterol and high triglyceride levels were independently associated with DPN and/or painful DPN [10].

Older age, longer duration of the diabetes and poor glycemic control are well-established risk factors for DPN and DPNP [7, 8, 11, 12]. Other reported risk factors are patients’ gender, height, high Body Mass Index (BMI), cigarette smoking, insulin therapy, alcohol consumption, elevated systolic blood pressure, presence of peripheral vascular disease, nephropathy, retinopathy and hypercholesterolemia [13–15].

Therapeutic success in most chronic illnesses including diabetes is often measured by disease free overall survival and control of major physical symptoms. However, metabolic measurements like glycemic control as well as clinical outcome measures such as pain can’t fully describe quality of life in DPNP. All these together with other psychological and emotional factors, as well as a person’s livelihood have influence on health status [16]. Hence management models that enhance patients’ health-related quality of life are important as they reduce non-compliance, and improve patients’ metabolic status, functional, psychological and social health and overall sense of well-being [17, 18].

Quality of Life (QoL) evaluation has emerged as an important outcome measure for chronic disease management. World Health Organization defines QoL as individuals’ perceptions of their position in life in the context of the culture and value systems in which they live and in relation to their goals, expectations, standards and concerns. On the other hand, Health Related Quality of Life (HRQoL) as one major domain of life, is also influenced by patients’ culture, ethnicity, educational level, income, disease-orientation and well-being [19].

Although many studies around the world have shown diabetes to have impact on QoL in terms of social, psychological and physical well-being, fewer studies are done on how DPNP is affecting the health domain of QoL (i.e., HRQOL) by itself alone [11, 20, 21]. Physical and mental components of QoL are significantly affected by painful DPN. Moreover, changes in physical components of QoL were associated with age and BMI, while poorer mental components of QoL were associated with female gender, smoking, BMI and diabetes duration. However, DPN alone had no statistically significant effect on either physical or mental QoL scores [10].

A study done in Korea showed that DPNP was independently associated with age, female gender, fasting plasma glucose, hypertension, and previous cerebrovascular events. Pain severity and interference measures were higher among patients with painful DPN than those without painful DPN, and patients with Painful DPN reported more sleep impairment and higher Visual Analog Score (VAS) [22] that measures the intensity of pain subjectively. A Croatian study conducted among DPN patients with and without neuropathic pain using SF-36 documented that patients with neuropathic pain had a statistically significant lower values compared to those without neuropathic pain in all 8 dimensions and both summary values [20]. A South African study using EuroQuol-5D (EQ-5D) Quality of Life Scale 3 questionnaire showed a statistically significant negative correlation between painful neuropathy and physical functioning, physical role limitation, social functioning, and pain domains [11].

To our best knowledge there is no study done in Ethiopia to examine HRQoL among patients with DPNP. This study is thus believed to shed light on potential interventions that improve diabetic patients’ HRQoL.

## Materials and methods

A facility based cross-sectional study with internal comparison was conducted in 2017 among randomly selected 110 type II DM patients with DPNP matched with 110 type II DM patients without DPNP in a 1:1 ratio, attending the same outpatient clinics of two hospitals in Addis Ababa, Ethiopia. DM patients between the age of 18 and 65 years, having DPNP for more than 6 months, those able to communicate, not in disabling pain and with no known abuse of alcohol participated in this study. The study received ethical clearance from the Addis Ababa University, College of Health Science, School of Public Health Research and Ethics Committee. Informed written consent was obtained from all study participants.

Sample size was determined using the logic of two independent mean for continuous outcome considering the HRQoL mean scores documented in a study done in South Africa [11]. The following assumptions were considered in the determination of sample size: mean score of 0.84 among those with DPNP and 0.64 among those without DPNP; 80% power; 1:1 ratio of patients with and without DPNP; precision value of 5% and 95% level of significance; and 10**%** non-response rate. Based on these assumptions, the total sample size calculated was 220, and this was allocated to the two hospitals based on the proportion of patient load in the hospitals.

### Measuring QoL

There are validated instruments to measure diabetes QoL as well as diabetic neuropathy [21, 22]. The Peripheral Neuropathy Quality of Life Instrument (PN-QOL-97) and Diabetic Specific Quality of Life Questionnaire (DSQOL) could not be used for this study as they are not validated in Ethiopia. Instead, we used the SF-36 questionnaire that was used and validated in two previous studies in Ethiopia [23, 24].

### Measurement

DPNP was determined through interview and physical examination using a standard neuropathic pain detecting tool (DouleurNeuropathique 4 questions (DN4)) [25], which is neuropathic pain diagnostic questionnaire. Intensity of pain was measured using the VAS score. Patients’ medical records were reviewed for presence of co-morbidities, DM related complication, Hemoglobin A1c (HbA1c), and lipid profile. Socio-demographic characteristics, duration of DM in year, medication modality, drug adherence as well as history of alcohol and drug use were obtained by interview using a structured questionnaire. HRQoL was measured using SF-36 questionnaire administered by researchers in a face to face interview.

The SF-36 questionnaire was selected as it can evaluate both the physical and psychological components of quality of life, and also its validity in studies related to neuropathy [26, 27]. SF-36 assesses eight dimensions of HRQoL, which relate to the physical and mental components of the individual’s health perception. These eight dimensions are ‘physical functioning’ (10 items), ‘role-physical’ which means role limitation due to physical health problems (4 items), ‘body pain’ (2 items), and ‘general health’ (5 items) more related to the physical component, ‘vitality/energy’ (4 items), ‘social functioning’ (2 items), ‘role-emotional’ which means role limitations due to emotional problems (3 items), and general ‘mental health’ (5 items). Each domain gets scores ranging from 0 (corresponding to the worst possible state) to 100 (corresponding to the best possible state). Furthermore, the two summary scores: the ‘physical component summary’ (PCS) that summarizes physical functioning, role-physical, body pain, and general health; and the ‘mental component summary’ (MCS) score that summarizes vitality, social functioning, emotional problems and mental health were calculated. All interviewed patients had undergone routine medical evaluation.

### Data analysis

Descriptive statistics was performed using SPSS version 21 to describe the socio-demographics, illness history, medication compliance, pain intensity as well as clinical, physical and biologic measurements as independent variables. Chi square and ANOVA test were applied to check variability between the two internally comparable groups (patients with and without DPNP) to enable us to control these factors in the model of analysis of HRQoL. The two groups were compared with regards to HRQoL by overall mean score, two summary scores as well as the eight individual domain mean scores. Since the outcome variable didn’t fulfill the normality assumption, the data was fixed by log transform and MANOVA was done to adjust other independent variables. Factor analysis based on 1993 Ware JE et.al. SF-36 Health Survey Manual and Interpretation Guideline [26] was performed to identify the relative influence of each domain for the overall HRQoL score. Correlation score analysis was also carried out to see the effect of pain intensity on HRQoL among patients with DPNP. Significant association between variables was declared if p <0.05.

## Results

### Socio-demographic characteristics of the study participants

One hundred thirty-two patients (60%) were female, 164 (75%) married, 62 (28.2%) completed high school education, 74 (34%) unemployed and 152 (69%) were followers of Orthodox Christian religion. The mean age was 50.4±9.9) years and mean monthly income was 58.6 USD. Chi square test demonstrated no significant variation between patients with and without DPNP in terms of basic socio demographic characteristics (see Table 1).

**Table 1:** Socio-demographic characteristics of type II DM^a^ patients, 2017.

Variables	Total	With DPNP^b^	Without DPNP n(%)	P- value
	n^c^ (%)	n(%)		
**Sex**				
Male	88(40)	47(42.7%)	41(37)	
Female	132(60)	63(57.3)	69(62.7)	0.49
**Age** (Mean, ±SD^d^)	50.4±9.9	52.7±9.3	47.4±10.6	0.28
**Educational Status**				
Illiterate	36(16.4)	21(19.1)	15(13.6)	0.53
Read and write only	31 (14.1)	16(14.5)	15(13.6)	
Primary school completed	52(23.6)	27(24.5)	25(22.7)	
Secondary school completed	62(28.2)	31(28.2)	31(28.2)	
College completed	39(17.7)	15(13.6)	24(21.8)	
**Marital status**				
Single	24(10.9)	10(9.1)	14(12.7)	0.59
Married	164 (74.6)	81(73.6)	83(75.5)	
Divorced	16 (7.3)	9(8.2)	7(6.4)	
Widowed	16 (7.3)	10(9.1)	6(5.5)	
**Religion**				
Orthodox	152(69.1)	78(70.9)	74(67.3)	0.82
Muslim	35 (15.9)	16(14.5)	19(17.3)	
Protestant	33 (15)	16(14.5)	17(15.5)	
**Occupation**				
Unemployed	75(34.1)	43(39.1)	32(29.1)	0.2
Government. employee	30 (13.6)	13(11.8)	17(15.5)	
Private organization employed	54 (24.6)	27(24.5)	27(24.5)	
Self employed	39(17.7)	14(12.7)	25(22.7)	
Retired	22 (10)	13(11.8)	9(8.2)	
**Mean income**	58.6	58.7	58.4	0.45

DM^a^–Diabetic Mellitus

DNPN^b^—Diabetic Peripheral Neuropathic Pain,

n^c^- number

SD^d^—Standard Deviation,

### Diabetes related characteristics of participants

One hundred ninety-six (88.7%) patients reported to adhere to their diabetic medications. The mean total cholesterol level was 213±28.2 mg/dl, (220±26.2 mg/dl for DPNP group, 205±28.7 mg/dl for those without DPNP). The mean duration of illness was 9.6̉±5.5 years (11.2±5.2 years among with DPNP, 7.5±5.4 years among without DPNP). The mean HbA1c level was 9.13±2.8% (10.4±2.7% for with DPNP, 7.5±2.7% for without DPNP).

BMI measurement showed that 107 patients (48.6%) were overweight, 31 (14%) obese and 76 (34.5%) had normal BMI. The mean BMI score was 25.9±3.7 (26.4±3.7 for patients with DPNP; 25.2±3.5 for those without DPNP). One hundred forty-two (64.5%) patients had one or more diabetes complications. Of these, 112 (78.9%) had neuropathy, 94 (66%) had hypertension, 30 (21%) had nephropathy, 19 (13.4%) had retinopathy, and 3 (2.1%) foot ulcer. In all the above variables, there was no statistically significant difference between those type II DM patients with and without DPNP.

ANOVA test for similarity between the two groups showed no significant difference in terms of total cholesterol level (p 0.246) and BMI (p = 0.615), whereas statistically significant difference was observed by duration of DM in years (p = 0.05) and HbA1c (p = 0.03).

### Diabetes and health related quality of life

As measured by SF- 36 eight components and the two summary scores, as well as the overall score, patients with DPNP had significantly poorer HRQoL than those without DPNP (p< 0.001). The highest mean difference was observed with role physical (39.1) followed by mental health and physical functioning at 38.2 and 30, respectively. Moreover, of the two summary scores, the mean difference for MCS was high compared to PCS, at 14.6 versus 9.3 (see Table 2).

**Table 2:** Mean score and mean sore difference among DM^a^ patients with and without DPNP, 2017.

HRQoL Domains	Mean (SD)			Independent samples T-Test for Equality of Means			
	Without DPNP	With DPNP	Total n = 220	Mean Difference	95% CI^b^		Sig. (2-tailed)
					Lower	Upper	
**PF**^c^	72.3(22.3)	42(19.1)	57.2(25.7)	30.3	24.6	35.8	.000
**RP**^d^	60.9(36.3)	21.8(28.2)	41.4(37.8)	39.1	30.5	47.7	.000
**BP**^e^	70.3(36.3)	45.6(15.5)	57.9(20.4)	18.5	14.6	22.4	.000
**GH**^f^	66(17.3)	39.9(16.5)	52.9(21.5)	18.7	14.4	23	.000
**VT**^g^	64.8(16.2)	46.3(13.1)	55.6(17.3)	17.6	13.2	22	.000
**SF**^h^	74.7(13.8)	57(19)	65.3(18.3)	24.8	20.5	29.1	.000
**RE**^i^	60.3(42.7)	22(33.3)	41.2(42.7)	26.1	21.5	30.7	.000
**MH**^j^	67.6(17.3)	48.9(15.1)	58.3(18.7)	38.2	28.0	48.4	.000
**PCS**^k^	47.4(7.7)	36(7)	41.7(9.3)	11.4	9.4	13	.000
**MCS**^l^	37.3(13.6)	22.6(11.6)	30(14.6)	14.7	11.4	18..1	.000
**Global**	67.2(18)	40(14)	53(21)	27	22.7	31	.000

DM^**a**^—Diabetic Mellitus

CI^**b**^—95% Confidence Interval

PF^**c**^–Physical Functioning

RP^**d**^—Role Physical

BP^**e**^—Body Pain

GH^**f**^—General Health

VT^**g**^—Vitality/Fatigue

SF^**h**^—Social Functioning

RE^**i**^—Role Emotion

MH^**j**^–Mentak Health

PCS^**k**^—Physical Component Score

MCS^**l**^—Mental Component Score

With regards to pain intensity, the mean score of VAS was 6.54(sd±0.95), ranging from 5 to 9. There was a statistically significant negative correlation between intensity of pain as measured by VAS score and the eight dimensions as well as the two summary scores of HRQoL (p <0.0001).

#### Mean score of health related quality of life

Mean score values for all the eight domains and the two summary scores of the SF-36 were uniformly better among patients who were younger, had a higher level of education, were single, had higher monthly income, had normal BMI, had less than 7% of HbA1c, had no DM complications and were using oral hypoglycemic agents as compared to their counterparts. Furthermore, higher mean score was documented for the PCS compared to that of MCS (18±31).

#### Multivariate Analysis of Variance (MANOVA)

MANOVA was performed to examine whether socio-demographic and clinical conditions influence the HRQoL as measured by PCS and MCS of SF-36. The result showed that only age has a statistically significant influence on both PCS and MCS (p <0.05). For in between group comparison, the post hoc test showed that patients in the age group of 51–60 years and 61–65 years had significantly lower HRQoL score compared to those in the age group of 20–50 years (P <0.05). Regarding the influence of clinical conditions, DPNP and DM related complication happened to have a statistically significant influence on both PCS and MCS (p<0.01). However, the duration of DM in years and HbA1C were found to have a statistically significant influence only on PCS. Those patients with illness duration less than five years had better quality of life compared to those above five years (see Table 3).

**Table 3:** Multivariate Analysis of Variance test for socio-demographics and clinical profile on HRQoL ^a^, 2017.

HRQoL		Socio-demographics on HRQoL					
		Age	Sex	Marital status	Educational Status	Occupation Status	Income
PCS ^b^	F	5.33	3.464	0.414	1.234	0.094	1
	P-value	0.001	0.067	0.743	0.304	0.984	0.397
MCS ^c^	F	2.57	0.482	0.442	1.516	0.82	2.34
	P-value	0.045	0.49	0.724	0.206	0.517	0.08
HRQoL		**Clinical profile on HRQoL**					
		DPNP^d^	DM complication	DM Duration (year)	HbA1c^e^	Types of Regimen	BMI^f^
PCS	F	7.449	6.612	4.142	3.336	1.645	1.777
	P-value	0.007	0.011	0.008	0.039	0.197	0.155
MCS	F	13.363	7.465	1.596	1.012	0.184	0.496
	P-value	0.000	0.007	0.194	0.367	0.832	0.685

HRQoL ^**a**^–Health Related Quality of Life

PCS b—Physical Component Score

MCS ^c^–Mental Component Score

DPNP^d^–Diabetic Peripheral Neuropathic Pain

HbA1C^e^–Hemoglobin A1c

BMI^f^–Body Mass Index

## Discussion

This study compared HRQoL among type II DM patients with and without diabetic peripheral neuropathic pain and the result showed a statistically significant mean score differences in all eight domains and the two summary scores of SF-36 between the two groups. This finding is similar with studies done in Turkey, Croatia, Korea and the UK [2, 25]. In another study, DPNP in type II DM patients independently affected both the physical and mental components of QoL, even after adjusting for the pain intensity [26].

In this study, higher mean difference was observed in MCS (14.6) compared to PCS (9.3), and among the eight dimensions, the highest mean difference was found with role physical domain (39.1) followed by mental health and physical functioning at 38.2 and 30 respectively. This finding is supported by a previous study done by Appich, M., J. John, et al (2008) which stated that PCS score is lower than MCS in each severity groups indicating that DPNP is affecting physical health more than mental health [27]. From the overall HRQoL score, the domain with the highest impact among all type II DM patients in our study was social functioning (score 65.3±18.8) while the least affected domain was role of emotion (score 41.2±42.7) and role of physical (41.4±37.8). The higher score in the social functioning domain than the rest might be explained by the strong social bondage and norms found in Ethiopia. This finding is consistent with the cross sectional study done among type II DM patients in UAE [28].

Depending on DPNP intensity, the Pearson correlation analysis of VAS score and HRQoL score showed statistically significant negative correlation in all domains of SF-36 (i.e. HRQoL scores decrease when the pain intensity increases). This finding is supported by studies done in different European countries which reported HRQoL decreased continuously with increasing DPNP severity [29, 30].

The multiple analyses of variance showed that age has a statistically significant negative effect on both mental and physical summary scores. People above 50 years of age have a statistically significant lower HRQoL score compared to people below 50 years. This finding is consistent with a study done in Turkey by Nurten Olmez YD et.al which reported significantly reduced quality of life in older age [30]. In our study, gender had no significant impact on the two summary scores of HRQoL. Similarly, a study done among type II DM reported comparable effect [31]. However, other studies reported men had a higher quality of life based on both summary values of SF-36 [20]. Regarding educational status, attaining a higher education was found to positively relate with HRQoL, and those who completed higher education had better HRQoL scores compared to those who are illiterate. This finding is similar to studies done in Argentina, Croatia, Kenya and Saudi Arabia [3, 4, 10, 30]. The main reason for this might be, as educational status increases people’s income and life style may be positively impacted, resulting in better quality of life.

Occupational status was also found to be associated with quality of life. In our study, those who are government and self-employee were found to have better scores on HRQoL domains particularly on physical functioning, social functioning and body pain domain. This finding is supported by earlier studies that indicated stable working enviroment and job security improved HRQoL among DM type II patients [31, 32].

DM duration and complication have statistically significant influence on HRQoL. Those patients who stayed diabetic for less than 5 years had higher mean score compared to those who stayed for more than 5 years. But there was no significant difference in HRQoL (Physical Health and Mental Health summary) means score among patients who stayed diabetic for 6–10, 11–15 and over 15 years. HbA1c was also one of DM related clinical variables which had a significant influence on PCS but had no impact on MSC score in this study. This finding is in line with previous studies which reported that prolonged disease duration and poor glycemic control are significant factors for poor HRQoL among type II DM patients [26, 31, 32]. Although, other studies suggested that BMI had negative effect on quality of life scores [4, 31], we didn’t find a statistically significant association between BMI and HRQoL in this study.

This study revealed the effect of DPNP on HRQoL among type II DM patients in Ethiopia. Besides filling the research gap, it encourages physicians to look for QoL among patients with neuropathic pain in general and subsequently implement appropriate interventions.

In conclusion, the present study found that DPNP is a major factor that influences various aspects of quality of life among type II diabetic patients in Ethiopia. Diabetic peripheral neuropathic pain had a great impact on the patients’ physical functioning, role of emotion, role of physical, which encompasses doing daily activities, self-care, income generation, social activities and emotional well-being.

Those patients with DPNP had significantly lower HRQoL as compared to those without DPNP and as the intensity of the pain increased, HRQoL significantly decreased. Other factors such as older age, long duration of DM, high HbA1c level, and DM complication had statistically significant negative influence on all domains of SF-36 HRQoL scores. Hence, health professionals should properly screen type II DM patients for DPNP and appropriate pain management should be provided in order to improve their HRQoL and survival.

## Conclusion

Health related quality of life of type II diabetic patients is negatively influenced by diabetic peripheral neuropathic pain, the common complication of diabetes. The Role Physical and Mental Health domains of health-related quality of life were the most impacted. Older diabetic patients, who suffered for longer duration of illness and those with diabetes related complications, were found to have poorer health related quality of life. Study using a combination of FS-36 with disease specific quality of life measurement is recommended. Furthermore, complimentary qualitative enquiry may also be required to explain issues such as why the role emotion is the list affected domain.

### Limitation of the study

However, the study may have limitations as laboratory diagnosis was taken from medical records which might have incomplete data. To address this gap, attempts were made to complete the data and exclude those records with lots of missing data. The study didn’t explore the association between all DM complications and HRQoL, which is a limitation. We recommend future studies to look into this. This study used only SF-36 as a generic tool. This is a limitation to the study that would have been more of informative if used in combination with a disease-specific tool.

## Supporting information

S1 Dataset (SAV)
